# eccDNAdb: a database of extrachromosomal circular DNA profiles in human cancers

**DOI:** 10.1038/s41388-022-02286-x

**Published:** 2022-04-06

**Authors:** Li Peng, Nan Zhou, Chao-Yang Zhang, Guan-Cheng Li, Xiao-Qing Yuan

**Affiliations:** 1 Guangdong Provincial Key Laboratory of Malignant Tumor Epigenetics and Gene Regulation, Guangdong-Hong Kong Joint Laboratory for RNA Medicine, Sun Yat-Sen Memorial Hospital, Sun Yat-Sen University, Guangzhou, China; 2 Medical Research Center, Sun Yat-Sen Memorial Hospital, Sun Yat-Sen University, Guangzhou, China; 3 Department of Research, The Affiliated Brain Hospital of Guangzhou Medical University, Guangzhou, China; 4 Guangdong Engineering Technology Research Center for Translational Medicine of Mental Disorders, Guangzhou, China; 5 Division of Functional Genome Analysis, German Cancer Research Center (DKFZ), Heidelberg, Germany; 6 Key Laboratory of Carcinogenesis of the Chinese Ministry of Health and the Key Laboratory of Carcinogenesis and Cancer Invasion of Chinese Ministry of Education, Cancer Research Institute, Central South University, Changsha, China; 7 Breast Tumor Center, Sun Yat-sen Memorial Hospital, Sun Yat-sen University, Guangzhou, 510120 China

**Keywords:** Epigenomics, Predictive markers

## Abstract

**E**xtra**c**hromosomal **c**ircular **DNA** (**eccDNA**) elements are circular DNA molecules that are derived from but are independent of chromosomal DNA. EccDNA is emerging as a rising star because of its ubiquitous existence in cancers and its crucial role in oncogene amplification and tumor progression. In the present study, whole-genome sequencing (WGS) data of cancer samples were downloaded from public repositories. Afterwards, eccDNAs were identified from WGS data via bioinformatic analyses. To leverage database coverage, eccDNAs were also collected by manual curation of literatures. Gene expression and clinical data were downloaded from TCGA and CCLE and then used to investigate the roles of eccDNAs in cancers. Finally, the first integrated database of eccDNAs, eccDNAdb, was developed. eccDNAdb currently includes 1270 eccDNAs, which were identified in 480 samples (of 42 cancers) after analyzing a total number of 3395 tumor samples (of 57 cancers) including patient tissues, patient-derived xenografts, and cancer cell lines. A total number of 54,901 eccDNA genes were annotated and included in the database as well. With the integration of gene expression, clinical information and chromatin accessibility data, eccDNAdb enables users to easily determine the biological function and clinical relevance of eccDNAs in human cancers. In conclusion, eccDNAdb is freely accessible at http://www.eccdnadb.org. To our knowledge, eccDNAdb is the first database in the eccDNA research field. It is expected to provide insight for novel cancer therapies.

## Background

Nuclear DNA is packaged into 23 pairs of linear chromosomes. Researchers have long observed the existence of **e**xtra**c**hromosomal **c**ircular **DNA** (**eccDNA**) elements [1, 2]. EccDNAs are derived from chromosomes but are independent of chromosomal DNA [3, 4]. Unlike chromosomal amplicons, eccDNAs follow a nonchromosomal mechanism of inheritance: because they lack centromeres, eccDNAs are segregated unequally into daughter cells; thus, daughter cells can have a higher eccDNA copy number than the parental cells [5, 6]. This unique mechanism of inheritance enables eccDNAs to change rapidly with the environment [6]. These observations demonstrate that eccDNAs undoubtedly exist, but these results were obtained before the advent of human genome sequencing technology. Computational analysis of high-throughput sequencing datasets offers a new perspective on eccDNAs in the genomic landscape of tumors.

Recently, through high-throughput sequencing, eccDNAs were found to exist more ubiquitously in human cancers, including glioblastoma, neuroblastoma, and breast cancer, than previously anticipated [4, 5, 7, 8]. eccDNA is a driver of eukaryotic genome plasticity and is involved in numerous biological processes [9]. Turner et al. noted that oncogene amplification, which drives tumor progression and intratumoral heterogeneity, is prevalent on eccDNAs [5]. Intratumoral heterogeneity induces chemotherapeutic resistance, and cancer cells can evade therapies that target oncogenes maintained on eccDNAs [10]. Moreover, eccDNAs drive the transcription of numerous oncogenes, including MYC, EGFR and CDK4, by increasing DNA copy numbers [7] and chromatin accessibility and enabling ultra-long-range chromatin contacts to promote cancer growth [4]. Two recent studies highlighted the association of eccDNAs with poor clinical prognosis in multiple cancers, including neuroblastoma [8, 11]. Clearly, the above studies indicated that amplification of eccDNA genes plays a crucial role in intratumoral heterogeneity, drug resistance and cancer progression. Revealing the underlying mechanism may broaden the horizon for targeted disruption of key oncogenes currently considered undruggable. Thus, eccDNA and eccDNA genes have great potential as treatment targets to prevent the progression and even the occurrence of cancers and as biomarkers for cancer diagnosis or prognostic prediction.

Thanks to this renewed understanding, there is an urgent need for a database to study the biological properties of eccDNAs and, more importantly, to facilitate eccDNA-based techniques for cancer treatment and prognosis prediction. Here, we developed the first integrated database of eccDNAs, eccDNAdb, which aims to identify known and novel eccDNAs in human cancers by computational analysis of whole-genome sequencing (WGS) data and to annotate and illustrate the potential roles of these eccDNAs in human cancers.

## Results

### Statistics of eccDNAdb data

**Fig. 1:**
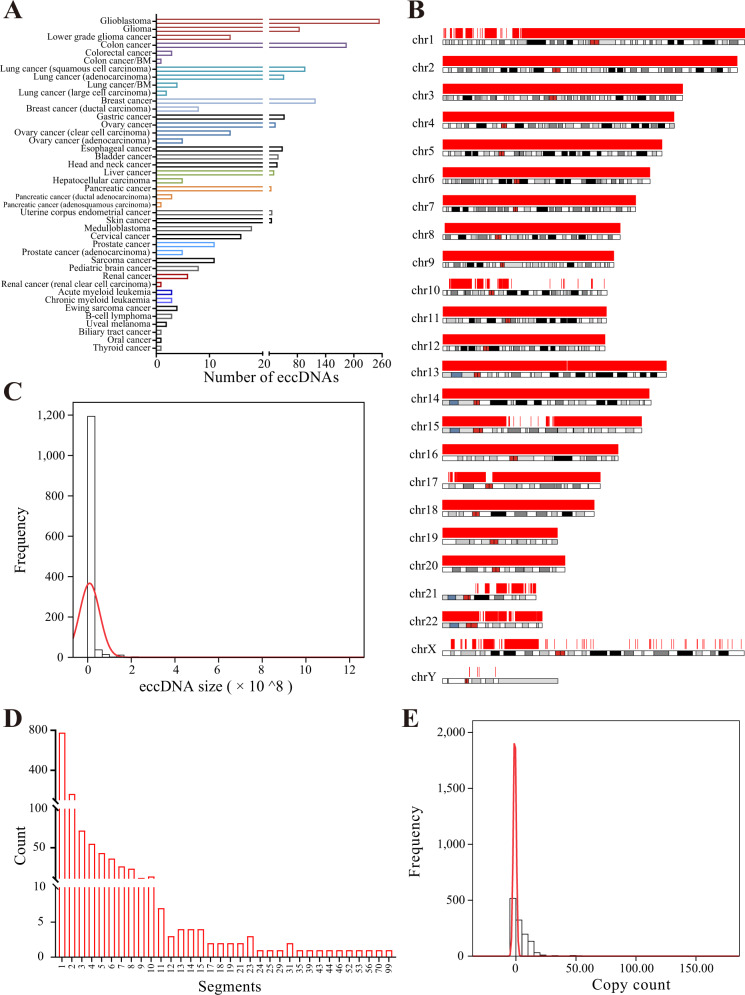
Statistics of eccDNAs in eccDNAdb. **A** Distribution of eccDNAs in human cancers. **B** Distribution of eccDNA segments in chromosomes. **C** Distribution of eccDNA sizes. **D** Distribution of eccDNA segment numbers. **E** Distribution of eccDNA copy counts.

In total, 1270 eccDNAs were identified, with 754 from WGS data and 516 from literature curation. EccDNAs were found in most studied cancers, demonstrating its ubiquitous existence in human cancers (Supplementary Table S2). Brain cancers, including glioblastoma, glioma, and lower grade glioma, had the most eccDNAs, while biliary tract cancer, oral cancer and thyroid cancer had the fewest. Most cancers had more than 5 eccDNAs (Fig. 1A). EccDNAs were identified on all chromosomes (Fig. 1B) and eccDNAs had a size ranging from 21 bp to 1 Gb (Fig. 1C). Approximately, one-fourth of the eccDNAs were <3.2 kb, half of the eccDNAs were <282 kb, and 75% of the eccDNAs were <2.5 Mb in size. The average eccDNA size was 9.4 Mb (Fig. 1C). The eccDNAs were composed of 1 to 99 segments, but most (61.26%) had only one segment (Fig. 1D). One-fourth of the eccDNAs were composed of three or more segments (Fig. 1D). For those eccDNAs identified from WGS data: eccDNA copy counts ranged from 1 to 160; mean copy count was 11.51; half of the eccDNAs had a copy count between 4 and 15; and nearly 30% of the eccDNAs had a copy count of <2 or >15 (Fig. 1E).

**Fig. 2:**
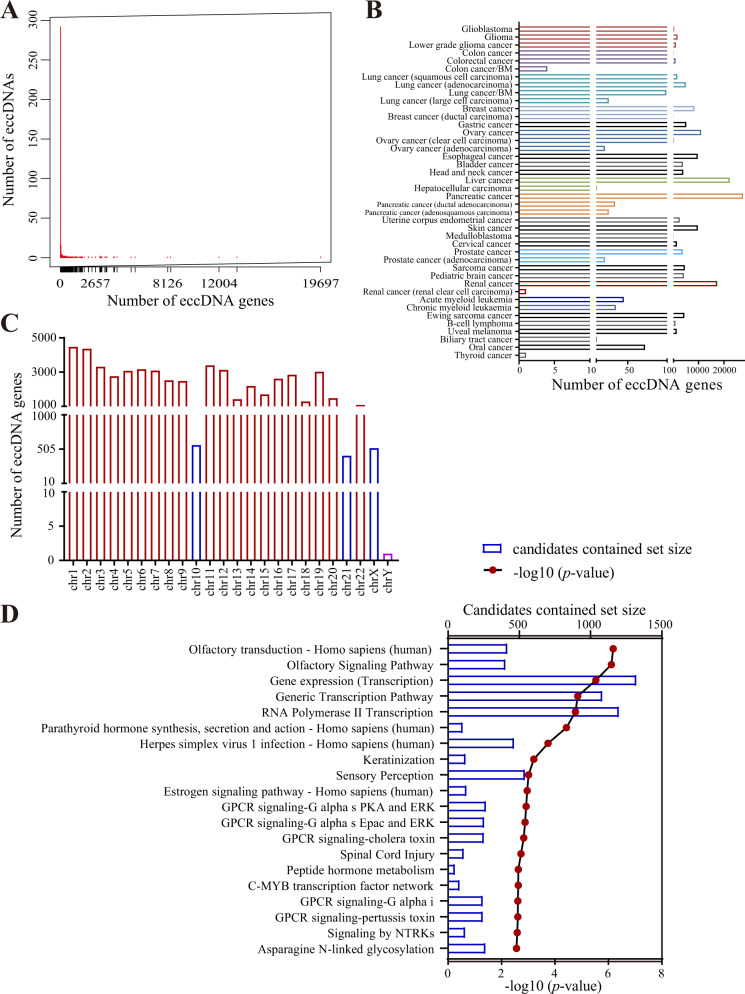
Statistics of eccDNA gene. **A** Frequency distribution relationship of eccDNA genes and eccDNAs. **B** Distribution of eccDNA genes in human cancers. **C** Distribution of eccDNA genes in chromosomes. **D** ConsensusPathDB pathway analysis of eccDNA genes in human cancers.

In total, 54901 genes were annotated as eccDNA genes (Supplementary Table S3). The number of genes varied among eccDNAs, with an average of 7 genes per eccDNA (Fig. 2A). Approximately 85.83% of eccDNAs had at least 1 gene (Fig. 2A). More than ten eccDNA genes were found in most cancers, except for colon cancer, renal cancer and thyroid cancer (Fig. 2B). The most eccDNA genes were identified in pancreatic cancer. EccDNA genes were distributed on all chromosomes (Fig. 2C; Supplementary Table S4). ConsensusPathDB [12] pathway analysis showed that these genes were enriched in biological processes including gene expression, transcription, estrogen signaling, GPCR signaling and so on (Fig. 2D). The distributions of eccDNA and eccDNA gene number by chromosomes were listed in Supplementary Table S5.

### Database description

**Fig. 3:**
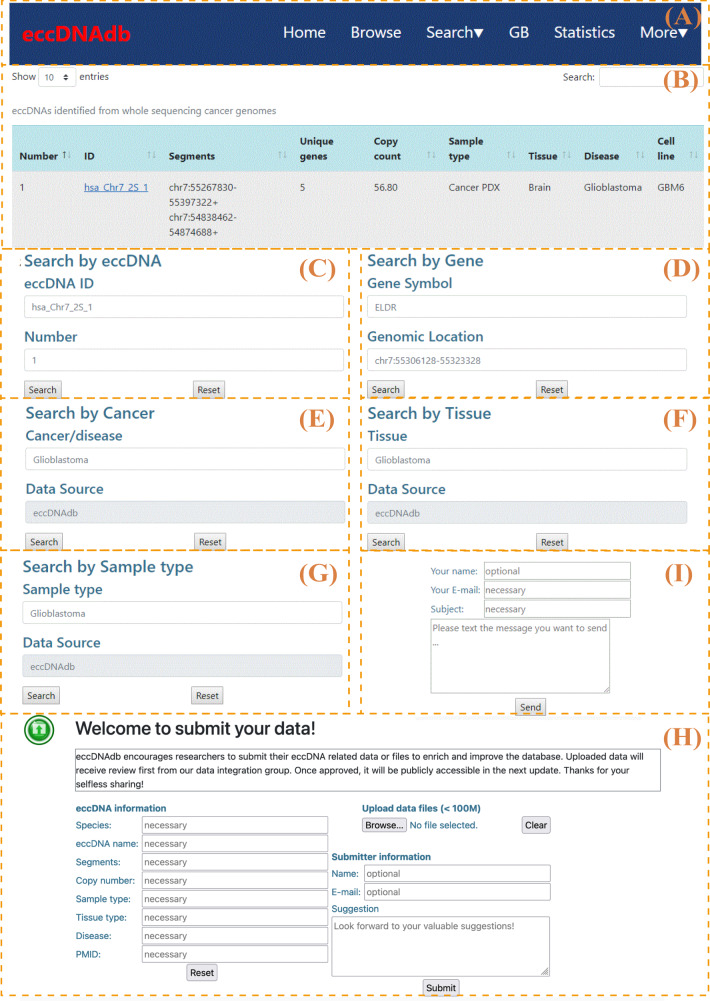
Overview of eccDNAdb. **A** Navigation bar. **B** “Browse” page of eccDNAs. **C**–**G** “Search” pages. **H** “Submit” form. **I** “Contact” form.

The database homepage provides a succinct introduction, a welcome message, and the development workflow. The navigation bar contains links for all resources in the database (Fig. 3A). On the “Browse” page, eccDNAs are listed in a table by row, and the information associated with the eccDNA is shown in the columns (Fig. 3B). In the top right corner of the table, there is a search box that can be used to filter content in the table. Clicking on the ID of an eccDNA will open its detail page, which is described in detail later in the “Database utility” section. The search process in eccDNAdb is straightforward, and various search strategies are provided (Fig. 3C–G). To submit data to eccDNAdb, the submit form on the submit page can be used (Fig. 3H). Regarding feedback, users are encouraged to send their suggestions to us via the feedback form on the contact page (Fig. 3I).

## Database utility

In this section, we mainly describe the “Browse”, “Search”, and “GB” modules and demonstrate how to utilize eccDNAdb with examples.

### Browse

All eccDNAs identified in this study can be accessed via the “Browse” link. In the data table on the “Browse” page, eccDNA entries are listed in rows, and eccDNA features are displayed in columns. Of note, a copy count of −1 in the “Copy count” column means this type of data is not available. The “ID” column shows the eccDNAdb ID of each eccDNA. The ID of an eccDNA is formatted as A_B_C_D, where (i) A is the origin of the species, (ii) B is the origin and number of the host chromosome(s), (iii) C is the number of segments, and (iv) D is the inclusion order of eccDNAs with the same designators for A, B, and C. Specifically, only data derived from humans were used in this study; thus, A is uniformly represented by “hsa”. B is represented by “Chr” plus a number or by a number plus “Chr”. The former format indicates that the eccDNA is derived from only one chromosome, and the number indicates the chromosome number; the latter format means that the eccDNA is derived from multiple chromosomes, and the number indicates the number of chromosomes. C is represented by a number plus “S”. “S” represents the word “segment”, and the number indicates the number of segments contained in the eccDNA. D is a random designator representing the order in which we assigned IDs to eccDNAs with the same designators for A, B, and C. D is always 1 if the A_B_C designator is not duplicated. Three eccDNA IDs are presented as examples: hsa_Chr20_6S_1 indicates that this eccDNA is derived from human chromosome 20 and consists of six segments; hsa_4Chr_9S_1 denotes a eccDNA that is derived from four distinct chromosomes and composed of nine segments; hsa_2Chr_8S_2 indicates that this eccDNA is derived from two human chromosomes, is composed of eight segments, and is the second eccDNA assigned the same hsa_2Chr_8S designator.

The eccDNA ID in the table is clickable. Clicking on the ID of an eccDNA opens its detail page. The information on the detail page is organized in separate sections. The “Basic information” section shows a basic description of the eccDNA, which is the same as the content on the “Browse” page.

After the “Basic information” section, structures of the eccDNA and amplicon are shown. The eccDNA structure is shown as a circular plot, and it also possesses interactive features. The inner circle of the plot shows the copy count of the eccDNA. The copy count appears when the mouse cursor moves on the layer. The outer circle shows the eccDNA segments and segment labels. Segment details will show up when moving mouse cursor onto it. A segment label is composed of a digital number and “>” or “<” symbols. The number indicates the segment order, and the “>” and “<” symbols denote the directions of the eccDNA segments. The directional symbols offer users a clear picture of how genomic segments are connected to form the eccDNA.

In the “eccDNA gene list” section, eccDNA genes are listed in a table. Clicking on a gene row will pick up the gene and show it in the following “eccDNA gene selector” panel. The “Pan-cancer eccDNA gene expression profile (TCGA)” and “Pan-cancer eccDNA gene expression profile (CCLE)” sections show the expression profiles of eccDNA genes in patient tissues and cancer cells, respectively. The “eccDNA gene expression in Tumor vs. Normal” section shows box plots of eccDNA genes’ expression in tumor vs. normal samples. In the box plot, *p*-values calculated by Student’s *t* test are shown in parentheses next to gene name on the x-axis. The “eccDNA gene in pan-cancer prognosis” section shows the survival analysis results for patients stratified by mean and median eccDNA gene expression levels. Near the bottom of the detail page, eccDNA gene-gene interaction network is shown in the section of “Interaction network of eccDNA genes”.

### Search

#### Search by eccDNA

**Fig. 4:**
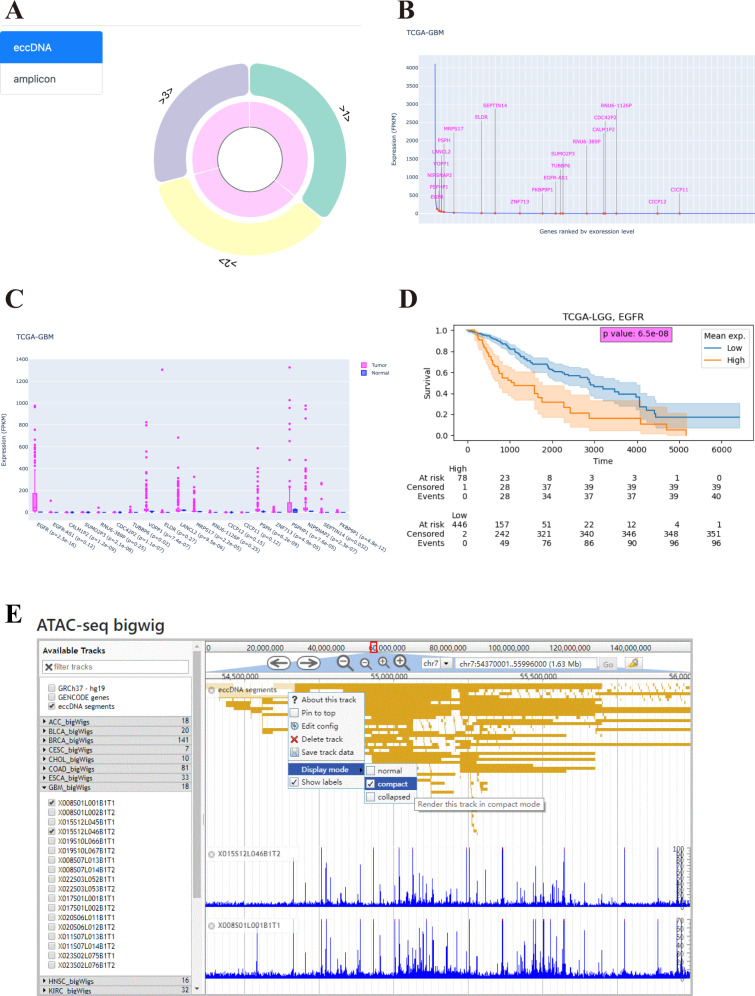
Introduction to eccDNA detail page. **A** Circular structure of hsa_Chr7_3S_1 in eccDNAdb. **B** Expression rank of hsa_Chr7_3S_1′s eccDNA gene in human glioblastoma (TCGA-GBM). **C** Comparison of hsa_Chr7_3S_1′s eccDNA gene expression between tumor and normal groups in glioblastoma (TCGA-GBM). **D** Overall survival differences between low grade glioma (TCGA-LGG) patients stratified by mean EGFR expression. **E** Chromatin accessibility of eccDNA-containing region chr7:54370001–55996000 in two glioblastoma samples.

We use “hsa_Chr7_3S_1” as an example to show the information provided by eccDNAdb. When “hsa_Chr7_3S_1” is inputted as a keyword, eccDNAdb returns available information about this eccDNA: “ID”, “Segments”, “Unique genes”, “Copy count”, “Sample type”, “Tissue”, “Disease”, and “Cell line”. When the link to this eccDNA is clicked, its detail page appears. On the detail page, we can see “Segments” as “1 chr7:55222713–55676816+ 2 chr7:55677806–56117062+ 3 chr7:54830972–55194959+” and “Disease” as “Glioblastoma” in the “Basic information” section. The eccDNA plot is shown following basic information (Fig. 4A). When the cursor moves onto a segment in the outer ring of the circular plot of eccDNA hsa_Chr7_3S_1, more information about this segment is revealed (Supplementary Fig. S1A). The inner ring shows the copy count (here, 100.25) of the eccDNA in glioblastoma (Supplementary Fig. S1B). The list of eccDNA genes belonging to hsa_Chr7_3S_1 is shown as Supplementary Fig. S1C. If the user is interested in the expression rank of any genes in a TCGA cohort (such as glioblastoma), the expression profile can be found in the “Pan-cancer eccDNA gene expression profile (TCGA)” section (Fig. 4B). Gene expression ranks in cancer cell lines can be found in the “Pan-cancer eccDNA gene expression profile (CCLE)” section (Supplementary Fig. S1D). Moreover, eccDNA gene expression differences between tumor and normal groups in TCGA cohorts can be found in the “eccDNA gene expression in Tumor vs. Normal” section (Fig. 4C). Further, we might wonder whether these eccDNA genes are associated with cancer prognosis. We can find that among the hsa_Chr7_3S_1 genes, high expression of EGFR indicates poor prognosis of LGG (Fig. 4D). Additionally, interactions of hsa_Chr7_3S_1 genes are visualized in the “Interaction network of eccDNA genes” section (Supplementary Fig. S1E).

#### Search by gene

On the page of “Search by Gene”, either gene symbol or gene location can be used to search eccDNAs. For example, entering ELDR in the search box would find all eccDNAs to which this gene belongs. In the result table, eccDNAs are listed by row, with columns showing ID, segments, unique genes, copy count, sample type, tissue, disease, and cell line. Clicking on an ID will open the detail page of indicated eccDNA.

#### Search by cancer

On the page of “Search by Cancer”, either cancer or disease name can be used to search eccDNAs. For example, entering “acute myeloid leukemia” in the search box would find all eccDNAs identified in this cancer. In the result table, eccDNAs are listed by row, with columns showing ID, segments, unique genes, copy count, sample type, tissue, disease, and cell line. Clicking on an ID will open the detail page of indicated eccDNA.

#### Search by tissue or sample type

On the “Search by Tissue or Sample Type” page, either the tissue name or sample type can be used to search eccDNAs. For example, entering “brain” or “cancer PDX” in the search box finds all eccDNAs in the corresponding tissue or sample. In the results table, eccDNAs are listed in the rows, with the columns showing ID, segments, unique genes, copy count, sample type, tissue, disease, and cell line. Clicking on an ID opens the detail page for the indicated eccDNA.

### Chromatin accessibility of eccDNAs

To determine the chromatin accessibility of eccDNA-containing genomic region, open chromatin regions across the genome were identified in assay for transposase-accessible chromatin with sequencing (ATAC-seq) data. ATAC-seq data were integrated, and a genome browser was provided for easy use of this feature. On the “GB” page, available ATAC-seq datasets are listed by cancer on the left. Clicking on the cancer name expands the viewport, and BigWig tracks are shown. The selected reference sequence, annotations, eccDNA segments, and ATAC-seq data are shown as tracks on the genome browser viewport. Users can use genome browser handlers to easily select and view the genomic region of interest.

We take EGFR and GBM as an example to describe how to use the “GB” feature. First, we check “GRCh37-hg19”, “ENCODE genes” and “eccDNA segments” boxes on the left of the genome browser. Then, we click “GBM_bigwigs” to expand this menu. After that, we input “EGFR” in the text box at the top of the genome browser viewport and click “Go”. The results show that chromatin is highly accessible at the EGFR gene locus and indicate its mapping eccDNA in GBM (Fig. 4E).

## Discussion

To our knowledge, eccDNAdb is the first integrated database of eccDNAs identified by the analysis of WGS data and literature curation. Interest in eccDNA elements is increasing because of their ubiquity and important roles in human cancers. EccDNAdb will facilitate eccDNA research in different ways. (i) A landscape of eccDNAs in human cancers is provided. The data we analyzed were derived from 3395 samples across 57 tumor types. (ii) The eccDNAs in eccDNAdb were carefully annotated. Database users can easily retrieve eccDNA structure, eccDNA gene, eccDNA gene expression profile, differentially expressed eccDNA gene, and cancer prognosis. This knowledge facilitates the understanding of why an eccDNA or eccDNA gene is important in cancer biology, thus providing insight for the development of novel cancer therapies. (iii) Database users can search eccDNAdb in multiple ways. Currently, searches by eccDNA, gene, cancer, tissue, and sample type are available. In addition, chromatin accessibility data of eccDNA-containing genomic regions in primary human cancers is provided in eccDNAdb. These features are very useful for finding the information that database users are interested in. Through large-scale WGS data analysis, eccDNAdb will allow a more comprehensive understanding of cancer etiologies from new perspectives and lay foundations for new therapeutics and impactful clinical trials.

EccDNAs exist more ubiquitously than expected in human cancers. They drive massive amplification of genes, leading to high gene expression. With an estimation of 19,292,789 new cases and 9,958,133 deaths in 2020 [13], cancer is a high incidence and leading cause of death worldwide. In the United States, ~1,898,160 cancer cases were expected to be diagnosed and 608,570 deaths from cancer were expected to occur in 2021 [14]. The overall clinical outcomes of patients are poor in many cancers and vary considerably among cancers and individuals. A recent study showed that oncogenes contained by eccDNA can predict unfavorable prognoses for cancer patients [8]. In eccDNAdb, associations between the overall survival of cancer patients and expression level of eccDNA genes are provided. Many eccDNA genes are indeed correlated with the clinical survival of cancer patients, as shown in our database.

ATAC-seq is a method for identifying open chromatin regions [15–19]. Corces et al. provided the genome-wide chromatinic accessibility profiles of 410 tumor samples across 23 cancer types from TCGA [20]. Chromatin accessibility, a hallmark of activated DNA modulation elements, plays a crucial role in linking the gene expression spectrum with epigenetic changes and ultimately influences the outcome of clinical treatment of cancer. EccDNA has been reported to exhibit higher chromatin accessibility and active chromatin state than chromosomal DNA, and this property is linked to higher transcription level of eccDNA [4]. In our database, the ATAC-seq data of 23 cancers were downloaded from the TCGA database. These data are shown on the “GB” page and combined with annotations of eccDNA and eccDNA genes. These integrated ATAC-seq and eccDNA data provide users with information on the chromatin regulatory landscape of eccDNA in primary human cancers.

Currently, there are four high-throughput methods for systematic identification of eccDNAs: WGS [4, 5, 7, 11], Circularization for In vitro Reporting of CLeavage Effects by sequencing (CIRCLE-seq) [21, 22], circular DNA enrichment sequencing (CIDER-Seq) [23] and ATAC-seq [24]. CIRCLE-seq is a technique for evaluating genomic off-target mutations induced by CRISPR-Cas9 editing [21, 22]. CIDER-Seq is a novel method to generate accurate eccDNA sequences and circular viral DNA sequences [23]. These two methods are anticipated to be rising stars for eccDNA research. However, a few sequencing data of these techniques are currently available. As mentioned above, ATAC-seq is usually used to identify open chromatin regions, and Kumar et al. found that it can detect eccDNA from tumor at the preamplification stage [24]. Unquestionably, the WGS technique has been used in more studies than other techniques and is reported in more scientific literature [4, 5, 7]. AmpliconArchitect is a robust and feasible tool for detecting eccDNA elements from WGS data [25]. Therefore, in this study, we systematically identified eccDNAs in human cancers by analysis of WGS data using AmpliconArchitect. Meanwhile, manual curation of eccDNAs from published articles was also performed to increase the database coverage.

In the future, we will continuously update eccDNAdb with newly published data and data submitted by users. Additional eccDNAs confirmed by low-throughput experiments or identified by other methods, such as CIDER-Seq [23], will be recorded in eccDNAdb to overcome current limitation that all eccDNAs in the database were identified by the AmpliconArchitect pipeline. In addition, more genetic and epigenetic information related to eccDNAs will be integrated. Numerous eccDNAs have been found in other organisms, including plants [26–28], yeast [29–31], kinetoplastids [32], *Saccharomyces cerevisiae* [33, 34], *Drosophila* [35], *Xenopus laevis* [36, 37], and *Mus musculus* [38]. Hence, more species and diseases will be considered in future updates.

## Conclusions

In summary, eccDNAdb, the first integrated database of eccDNAs, is a comprehensive catalog of eccDNAs identified in human cancers by computational analysis of WGS data and by manual literature curation. EccDNA genes were identified and annotated with expression data from TCGA and CCLE databases. The database provides not only basic information about eccDNAs but also the prognostic value of eccDNA genes. Meanwhile, ATAC-seq data from TCGA were integrated for the exploration of eccDNA’s chromatin accessibility. It is expected that the database will deepen our understanding of the mechanisms of eccDNAs in cancer development and facilitate eccDNA research.

## Materials and methods

### Data collection

WGS data of 183 tumor samples and 8 normal samples (Supplementary Table S1) were downloaded from the NCBI SRA database. First, low-coverage (<5×) WGS data of 131 samples (123 tumor and 8 normal), originally generated by Turner et al. [5] were downloaded from SRA study SRP081035. Second, high-coverage (>5×) WGS data of 44 tumor samples were collected and downloaded from SRA. To find high-coverage data for different types of human cancers, a reference list of cancer types was created according to the major primary cancer sites included in the TCGA database, and the SRA database was then searched with the reference cancer types as a search term. Considering biological significance and computation speed, no more than three high-coverage SRA runs per cancer type were downloaded and analyzed in this study. For cancers that had more than three SRA runs, three runs were randomly selected for analysis. Third, WGS data of 16 samples of cancer cells generated by the CCLE project (PRJNA523380) were downloaded from SRA. Finally, the low- and high-coverage WGS data and CCLE data were combined into a single WGS dataset for further analysis.

In order to make our database comprehensive, we also performed manual curation of literatures to collect eccDNAs that are identified using AmpliconArchitect and readily available, in addition to ab inito analysis of WGS data. Eventually, we collected 516 eccDNAs from the article published by Kim et al. [8] who generated the results after analyzing 3212 patient samples (Supplementary Table S1).

### EccDNA identification from WGS data

WGS reads were mapped to the human reference genome hg19 with BWA [39]. Copy number variations (CNVs) were identified with ReadDepth using default parameters [40]. Notably, sex chromosomes were eliminated from the analysis because ReadDepth regards them as entirely deleted in males. CNVs with copy number >5 and size >100 kb were selected and merged into seed intervals with amplified_intervals.py, and amplicons were further identified with AmpliconArchitect.py in the AmpliconArchitect software suite [25]. We considered copy number >5 by referring to Deshpande et al.’s study who investigated CNV of matched tumor and normal cases from TCGA and found this as the criterion to identify somatically amplified intervals [25]. Default settings of AmpliconArchitect were used during the analysis. After amplicon detection, eccDNAs were extracted from cycle files by converting amplicon cycle data into eccDNA data via in-house scripts. Because a cycle in the cycle file can also be reconstructed without the determined endpoint, such a cycle is in fact a linear contig and will not be selected as an eccDNA.

Normal samples were used as a control for the detection of eccDNA in tumors. CNVs of eight normal samples were obtained. The set of amplified intervals for normal samples were created and then marked as blacklisted regions. Afterwards, tumor intervals overlapping blacklisted regions were trimmed to exclude the portions within 1 Mbp of the blacklist regions [25].

### Annotation of eccDNA genes

Genes that entirely or partially overlap with one or multiple eccDNAs are defined as eccDNA genes. To identify eccDNA gene, comprehensive gene annotation was downloaded from the GENCODE database (GRCh37 version of release 38). Then, BedTools intersect was used to find overlaps between eccDNA and GENCODE gene [41]. Overlap size, namely the number of intersecting bases, was recorded and subsequently used to calculate the overlap ratios for eccDNA and gene, respectively. The eccDNA ratio (*R*_*e*_) was calculated as the overlap size divided by the eccDNA size, i.e., $R_e \,=\, \frac{{Overlap\,size}}{{eccDNA\,size}}$, while the gene ratio (*R*_*g*_) was calculated as the overlap size divided by the gene size, i.e., $R_g \,=\, \frac{{Overlap\,size}}{{Gene\,size}}$. Overlap ratio ranges between 0 and 1, with 0 indicating no intersection between two queried features while 1 indicating one feature entirely overlaps with the other. For example, a gene ratio of 1 means this gene completely resides in the queried eccDNA.

### Expression of eccDNA genes

To examine the expression of eccDNA genes in human cancers, data for gene expression in patient samples and cancer cells were downloaded from TCGA via the GDC Data Portal (https://portal.gdc.cancer.gov/) and CCLE (https://portals.broadinstitute.org/ccle), respectively. EccDNA gene expression was analyzed at two levels: (i) a detailed expression profile of eccDNA genes in each cancer; and (ii) a comparison of gene expression levels between normal and tumor samples. Two-tailed *t* test was performed in Python to determine differences between the normal and tumor groups.

### Survival analysis

To analyze the prognostic value of eccDNA genes in human cancers, clinical information was also downloaded from TCGA via the GDC Data Portal (https://portal.gdc.cancer.gov/). The mean or median expression levels of eccDNA genes were used to stratify cancer patients. Given an eccDNA gene, patients with an expression level below the mean or median value were defined as the low expression group, and those with an expression level above the mean or median value were defined as the high expression group. The lifelines library in Python was then used to analyze the effect of eccDNA genes on cancer prognosis.

### Database implementation

The database was developed with modern web programming languages such as HTML5 and CSS3. Bootstrap v4 (https://getbootstrap.com/) was used to design the layout of the user interface. DataTables (https://datatables.net/) was used to show tabular data on the webpages. jQuery (https://jquery.com/) was used for enhanced interactive functionality. The circosJS (https://github.com/nicgirault/circosJS) library was used to visualize eccDNA structures on the webpage. The Plotly graphing library (https://plotly.com/) was used for dynamically rendering graphs on the webpage. Data were stored in an SQLite (https://www.sqlitetutorial.net/) database. The STRING database API was used to construct interaction network of eccDNA genes [42]. The web application was developed with the Django (http://djangoproject.com/) framework. The website was deployed in the Alibaba Cloud in an ECS host running Ubuntu 18.04 and Apache2.

## Supplementary information

Legends of supplemental Figure S1 and Table S1-S5

supplemental Figure S1

supplemental Table S1

supplemental Table S2

supplemental Table S3

supplemental Table S4

supplemental Table S5

## Data Availability

All data generated or analyzed during this study are included in this published article and its Supplementary Information files.

## Code availability

The source code for the identification of eccDNAs and for the annotation of eccDNA genes is available at https://github.com/solo7773/eccDNAdb.
