# Supplementary material for: eccDNAdb: a database of extrachromosomal circular DNA profiles in human cancers
Source: Oncogene. 2022 Apr 6;41(19):2696–705. doi: 10.1038/s41388-022-02286-x (PMC9076536; doi:10.1038/s41388-022-02286-x)
Supplement: Supplementary file 1 — Legends of supplemental Figure S1 and Table S1-S5 [file 41388_2022_2286_MOESM1_ESM.docx]

**eccDNAdb: a database of extrachromosomal circular DNA profiles in human cancers**

**Supplementary information**

**Supplementary Figure S1. An eccDNA example to show the information provided by eccDNAdb.** (A) eccDNA gene name in the outer circular map of one eccDNA, hsa_Chr7_3S_1, provided in “eccDNA” of eccDNAdb. (B) Copy count in the inner circular map of eccDNA hsa_Chr7_3S_1 in glioblastoma. (C) Information on one example segment (segment 2 of eccDNA hsa_Chr7_3S_1), including “Segment”, “Coordinate”, “Direction” and “eccDNA genes” (for “eccDNA genes”, “Name”, “Coordinate” and “Oncogene” are included on the “Segment details” page). (D) Expression rank of eccDNA (hsa_Chr7_3S_1) genes in CCLE. The central nervous system (CNS) cell line 42MGBA is used as an example. (E) Interaction network of eccDNA hsa_Chr7_3S_1 genes.

**Supplementary Table S1**. Information on the samples that have been used for eccDNA detection in this study.

**Supplementary Table S2**. Information for eccDNAs in eccDNAdb.

**Supplementary Table S3**. Information for eccDNA genes in eccDNAdb.

**Supplementary Table S4**. The distribution of eccDNAs and eccDNA genes in human cancers from eccDNAdb.

**Supplementary Table S5**. The distribution of eccDNAs and eccDNA genes on human chromosomes from eccDNAdb.
