# Supplementary material for: eccDNAdb: a database of extrachromosomal circular DNA profiles in human cancers
Source: Oncogene. 2022 Apr 6;41(19):2696–705. doi: 10.1038/s41388-022-02286-x (PMC9076536; doi:10.1038/s41388-022-02286-x)
Supplement: Supplementary file 2 — supplemental Figure S1 [file 41388_2022_2286_MOESM2_ESM.pdf]

Supplemental Figure S1

A

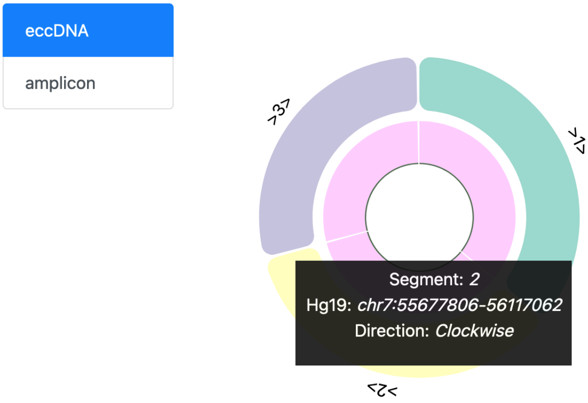

D

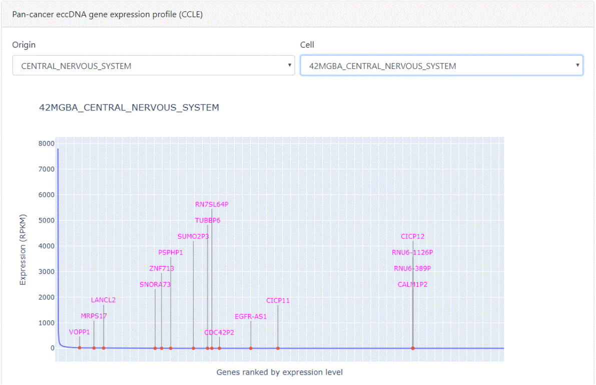

B

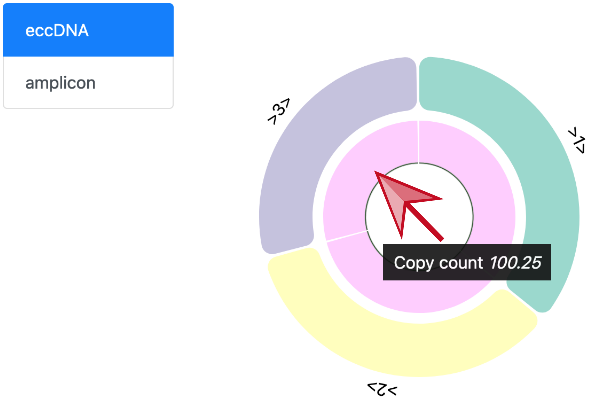

E

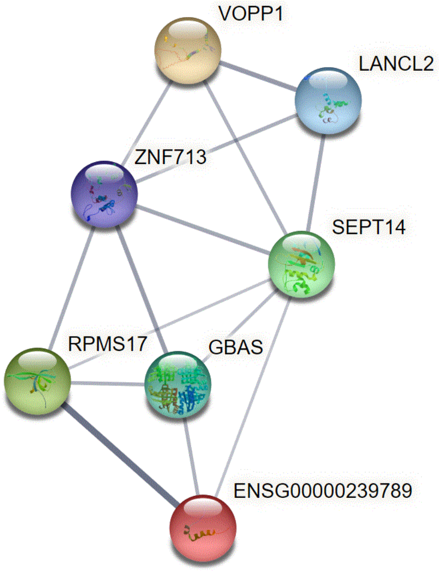

C

eccDNA gene list

Show 10 entries Search:

eccDNA segment	Gene	Gene locus (strand)	Overlap size	eccDNA ratio	Gene ratio	Ensembl gene	Gene typ
chr7:55677806-56117062	RP11-310H4.6	chr7:55724461-55746183 (-)	21722	0.049	1	ENSG00000231317.1	ui
chr7:55677806-56117062	FKBP9P1	chr7:55748767-55780945 (-)	32178	0.073	1	ENSG00000176826.15	tr
chr7:55677806-56117062	SEPTIN14	chr7:55861233-55930448 (-)	69215	0.16	1	ENSG00000154997.9	pi
chr7:55677806-56117062	NIPSNAP2	chr7:56019486-56067874 (+)	48388	0.11	1	ENSG00000146729.10	pi
chr7:55677806-56117062	PSPHP1	chr7:55832490-55840981 (+)	8491	0.019	1	ENSG00000226278.1	ui
chr7:55677806-56117062	ZNF713	chr7:55955149-56010223 (+)	55074	0.13	1	ENSG00000178665.16	pi
